# Mesenchymal Stem Cells and Their Role in Neurodegenerative Diseases

**DOI:** 10.3390/cells13090779

**Published:** 2024-05-02

**Authors:** Vincenzo Mattei, Simona Delle Monache

**Affiliations:** 1 Department of Life Science, Health and Health Professions, Link Campus University, 00165 Rome, Italy; v.mattei@unilink.it; 2 Department of Biotechnological and Applied Clinical Sciences, University of L’Aquila, 67100 L’Aquila, Italy

Mesenchymal stem cells (MSCs) have garnered significant interest in the field of regenerative medicine for their ability to potentially treat various diseases, especially neurodegenerative disorders [1]. These cells are highly adaptable, and have the capacity to differentiate into diverse cell types, making them ideal candidates for cell-based therapies [2,3]. Preclinical studies have displayed promising results utilizing MSCs to address neurodegenerative conditions such as Alzheimer’s, Parkinson’s, and Huntington’s diseases. These diseases are characterized by the progressive degeneration of neurons, leading to cognitive and motor deficits [4]. MSCs have shown the potential to modulate the inflammatory response in Alzheimer’s disease, thereby decreasing neuroinflammation and fostering neuron survival. Additionally, MSCs can assist in clearing amyloid-beta plaques, a hallmark of Alzheimer’s disease, through their immune-modulating properties. In the case of Parkinson’s disease, MSCs may potentially differentiate into dopamine-producing neurons, which are typically lost in this disorder, and integrate into the brain to restore neuronal function. Moreover, MSCs can release neurotrophic factors [5] that promote the growth and viability of existing neurons in the brain. Also, mesenchymal stem cells (MSC) have shown promising potential in the treatment of epilepsy due to their anti-inflammatory properties and ability to promote neurogenesis. Epilepsy is a neurological disorder characterized by recurrent seizures. MSCs exert their therapeutic effects through various mechanisms, including immune modulation and neuroprotection. They regulate the immune response by inhibiting inflammatory cytokines and promoting anti-inflammatory cytokines, while also protecting neurons from damage, reducing oxidative stress, and promoting cell survival. Clinical evidence has demonstrated the efficacy of MSCs in epilepsy treatment, with studies showing a reduction in seizure frequency and improved cognitive function in epilepsy patients.

Tesiye and colleagues explore the topic of epilepsy, a life-threatening neurological disease that impacts around 70 million individuals globally. While some patients can effectively manage their condition with currently-used antiseizure medications (ASMs), approximately 30% of epilepsy patients require alternative therapies due to pharmaco-resistance [6,7]. In their review, Tesiye et al. documented the impacts of ASMs on stem cell therapy when co-administered, and also highlighted recent advancements in the study of mesenchymal stem cells (MSCs) in both pre-clinical and clinical research. Indeed, isolation of these cells from allogeneic and autologous sources, followed by transplantation into the body, has displayed significant therapeutic potential in a variety of epilepsy models [8]. Tesiye et al. observed that these cells may be a potent tool to address epilepsy and overcome several limitations of current therapies. This is attributed to their ability to easily find their way to the affected tissue, interact with the nervous system, and continuously regenerate themselves over an extended period of time. Moreover, they observed the release of a variety of trophic and immunity factors, in addition to different neuromodulators and neurotransmitters, and the ability to deliver therapeutic molecules, which has made them reliable therapeutic tools [9]. It seems like Tesiye et al. found that treatments with MSCs led to an improved seizure threshold, shorter seizure duration, and decreased frequency and strength of epileptic discharges. This suggests that MSCs could have a potential positive impact on epilepsy treatment [10]. Despite the promising results, further studies are needed to fully understand the benefits of stem cell therapy for epileptic patients and how it interacts with current treatments. Future research should focus on optimizing MSC delivery methods and understanding the long-term effects of MSC therapy. In conclusion, MSCs hold great promise in the treatment of epilepsy because of their anti-inflammatory and neuroprotective properties. Further investigation is necessary to fully exploit their therapeutic potential.

Candelise and colleagues emphasized the significance of utilizing stem cells isolated from human dental pulp and exfoliated deciduous teeth as a potential therapeutic approach for nervous system pathologies. Despite extensive research over the years, there are still no effective therapies available to slow down or stop the progression of neuro-degenerative disorders. Cell therapy has emerged as an intriguing alternative to the classical pharmacological approach. In particular, in recent years, the transplantation of adult stem cells could be both relevant and important, as they can be obtained from various adult tissues without major concerns [11]. In their review, the authors discussed the progress made in utilizing stem cells in preclinical models of neurodegenerative disorders, outlining the differences and similarities in their characteristics, differentiation abilities, and potential clinical applications. The authors found that stem cells isolated from human dental pulp and exfoliated deciduous teeth are particularly promising for treating neurodegenerative conditions. These cells, derived from the neural crest, have the capacity to develop into different types of mature neurons [12,13]. Additionally, their easy extraction process compared to other types of mesenchymal stem cells is a significant advantage. The authors also highlighted other advantages of using these stem cells, such as the ability to perform autologous transplants, which avoids ethical concerns and reduces the risk of rejection. These cells have shown promise in improving various forms of neurodegenerative disorders [14], and have demonstrated the ability to cross biological barriers and migrate to the affected brain regions, where they can differentiate. Furthermore, the collection of these stem cells could potentially lead to the creation of stem cell biobanks, storing renewable cells for future autologous grafts and significantly lowering the risk of rejection during transplants.

Minoia and colleagues have highlighted the strong connection between bone health and brain function, noting that changes in one system can impact the other. As the population ages, the link between bone health and neurodegenerative diseases becomes more apparent [15,16,17]. Research has shown that both the central and peripheral nervous systems play a crucial role in bone remodeling [18,19], with various sensory neurotransmitters influencing the aging process and osteogenic differentiation, leading to skeletal issues such as osteoporosis and arthritis. Understanding the intricate relationship between bones and nerves is essential for developing new treatments for bone disorders and inflammatory conditions, which could potentially slow down the progression of neurodegenerative diseases. While extensive studies have been conducted to unravel the causes of neurodegenerative disorders, many of the molecular mechanisms contributing to these diseases remain unknown. The authors propose that exploring the bidirectional communication between bones and nerves could uncover novel therapeutic strategies. Although the current experimental models are limited in their ability to study the complex interactions between the brain and bones, advancements in techniques such as 3D modeling offer promise for future research. Additionally, studies have shown the beneficial effects of microvesicles and exosomes released from mesenchymal stem cells in restoring neuronal cell function by crossing the blood–brain barrier. Nanoparticles are also being investigated for their potential to deliver drugs or biological molecules to the central nervous system, offering new possibilities for treating conditions such as Alzheimer’s disease with targeted therapies. In conclusion, the use of nanoformulations that target specific tissue markers presents a promising avenue for developing therapeutic interventions for various diseases, including neurodegenerative disorders.

Zayed and colleagues delve into the topic of prion diseases, which are neurodegenerative disorders characterized by being progressive, incurable, and fatal. The cellular form of prion protein (PrP^C^) is a highly conserved cell surface GPI-anchored glycoprotein that was identified in cholesterol-enriched, detergent-resistant microdomains named “rafts” [20]. The prion consists of PrPSc, the misfolded pathogenic isoform of the cellular prion protein (PrP^C^). Recent studies have highlighted the crucial role of PrP^C^ in various physiological processes, such as cellular differentiation, proliferation, adhesion, and neural development. Indeed, PrP^C^, whose expression is modulated according to the cell differentiation degree, appears to be part of the multimolecular signaling pathways of the neuronal differentiation process [21]. Additionally, studies report that prion protein is expressed on the membrane surface of a variety of stem cells (SCs), where it plays an important role in the pluripotency and self-renewal matrix, as well as in SC differentiation [22,23]. Zayed reported that SCs can propagate the pathogenic form of the prion protein, making them a potential model for studying prion diseases in vitro. Furthermore, due to their capability to self-renew, differentiate, immunomodulate, and regenerate tissue, SCs hold promise for being used in the treatment of various neurodegenerative disorders, including prion diseases. The authors summarize various preclinical studies that have explored the use of stem cells in treating prion diseases [24,25,26,27]. Using SCs as a treatment strategy shows great potential for regenerating damaged cells and improving clinical outcomes. Several preclinical trials used SCs derived from bone marrow (BM), and other SCs have been utilized as transplants to repair damaged neural cells in the brains of prion-induced mice. While some results have been promising, the efficacy of stem cell therapy in prion diseases is still under investigation, and careful consideration of the type and source of stem cells used in research is necessary.

## Conclusions

In this Special Issue, some papers have been collected in which the potential role of MSCs in neurodegenerative diseases is explored. MSCs seems to have therapeutic potential in the treatment of epilepsy and other neurodegenerative diseases, such as prion diseases, and in the modulation of bone tissue in relation to the nervous system. In the future, more efforts will be needed to apply MSCs to neurodegenerative diseases.
